# *Candida* Species in Children Undergoing Orthodontic Treatment with Removable Appliances: A Pilot Study

**DOI:** 10.3390/ijerph20064824

**Published:** 2023-03-09

**Authors:** Aleksandra Brzezińska-Zając, Magdalena Sycińska-Dziarnowska, Gianrico Spagnuolo, Liliana Szyszka-Sommerfeld, Krzysztof Woźniak

**Affiliations:** 1 Department of Orthodontics, Pomeranian Medical University in Szczecin, Al. Powst. Wlkp. 72, 70-111 Szczecin, Poland; 2 Department of Neurosciences, Reproductive and Odontostomatological Sciences, University of Naples “Federico II”, 80131 Napoli, Italy; 3 School of Dentistry, College of Dental Medicine, Kaohsiung Medical University, Kaohsiung 80708, Taiwan

**Keywords:** *Candida* species, *Candida albicans*, orthodontic treatment in children, removable orthodontic appliance, oral health during orthodontic treatment

## Abstract

The purpose of this study was to analyze the effect of orthodontic treatment with removable appliances on the growth of *Candida* spp. in children undergoing orthodontic treatment. The study included 60 patients of equal numbers as to gender from the orthodontics department of the Pomeranian Medical University in Szczecin, Poland. All patients were aged 6–12 years and were qualified for orthodontic treatment with removable appliances. The following examinations were performed on the day of treatment initiation (T1) and 6 months after the start of treatment (T2); a collection of oral swabs for culture on Sabouraud’s medium and the identification of fungal colonies using the VITEK^®^2 YST. At T1, 42 (70%) subjects, were free of *Candida*, while after 6 months of treatment, the number decreased to 25 (41.67%). Two types of fungi, *C. albicans* and *C. parapsilosis*, predominated in the test performed at T1. The study at T2 showed that *C. albicans* most frequently colonized the oral cavity in 23 children (38.33%). Three new strains *C. dubliniensis*, *C. kefyr*, and *C. krusei* were identified at T2. Statistical analysis showed a significant correlation between the culture results and the age of the patient at T2. Patients older than 9 years had significantly more positive tests. Orthodontic treatment with removable appliances contributes to increased oral colonization by *Candida* spp.

## 1. Introduction

Removable orthodontic appliance treatment is a popular method of orthodontic therapy among young, growing patients. The undoubted advantages of removable appliances include the ease of maintaining hygiene, both of the appliance and of the entire oral cavity. There are no restrictions on access to the tooth surface during cleaning. In adolescent patients, who quite often struggle to maintain proper oral hygiene, this is an important factor in favor of treating malocclusion with removable devices [1,2]. On the other hand, orthodontic treatment with removable devices is often a rather uncomfortable process. For the youngest patients, appliances are perceived as “foreign bodies”, placed in a highly sensitive area. This can result in difficulties in accepting the plate, both for physiological reasons such as discomfort while wearing it or difficulties in activities previously performed subconsciously such as swallowing saliva, as well as for psychological reasons such as questioning of the appearance and the effect on the child’s pronunciation during treatment [3].

Removable orthodontic appliances are made up of several components, among which the acrylic plate that makes up the majority of the surface of the appliance is the part that most affects the oral microbiota. The acrylic plate is considered a major risk factor for allergic lesions and causing fungal infections in the oral cavity. The substance included in the acrylic plastic used in orthodontics is methacrylic acid methyl ester (methyl methacrylate monomer, MMA-C5H8O2). According to the classification of the substance in accordance with Annex VI of the Regulation of the European Parliament and of the Council of 16 December 2008, MMA is classified as highly flammable, irritant to the skin and respiratory tract, and sensitizing upon skin contact. However, it is not considered a human carcinogen [4]. At the end of the polymerization process, depending on the conditions under which it is carried out, between 0.2% and 7% of free methyl methacrylate, the so-called residual monomer, remains in the appliance. It is in methacrylate that most of the causes of sensitization reactions are attributed which can lead to changes in the oral microflora. In recent years, opportunities have been sought to reduce the number of *Candida* species in removable appliances. In a study by Shahabi et al., the addition of chitosan nanoparticles (NPs), known for their antimicrobial properties, to cold-cured acrylic resin had no significant negative effect on its flexural and compressive strength [5]. 

A healthy oral cavity provides a natural habitat for a diverse group of microorganisms. The ecological niches are primarily bacteria, fungi, protozoa, or mycoplasmas, and in some cases, viruses are also identified. All of them together mainly inhabit the gastrointestinal tract, including just the oral cavity, which is its first section and forms the endogenous microflora, also known as the microbiota [6]. The discovery of the ability of host cells and bacterial cells to communicate with each other proves the existence of a network of relationships forming one large ecosystem that can determine the health and disease of the human body [7,8]. Dworecka-Kaszak [9] noted the occurrence of 74 species of fungi that could be grown under laboratory conditions. Among them, *Candida* (75%), *Cladosporium* (65%), *Aureobasidium* (50%), *Saccharomyces* (50%), *Aspergillus* (35%), *Fusarium* (30%), and *Cryptococcus* (20%) predominated. The microbiome located in the oral cavity is characterized by a fairly high stability in the abundance of individual groups of microorganisms so that the individual microbial species present there coexist with each other under conditions of homeostasis. The qualitative composition of the human microbiome is more complex and individually diverse than initially thought. It is subject to development and constant change. The microbiome is influenced not only by diet or the type and quality of food consumed but also by age, cultural and social conditions, and genetic and environmental factors [10].

Yeast-derived fungi, which include all of the *Candida* species, have not been clearly classified as strictly pathogenic. Their importance in oral biocenosis has not yet been clearly explained. Some authors believe that they are present in the healthy oral cavity as commensal microorganisms, and thus are part of its physiological microflora. On the other hand, according to other researchers, *Candida* fungi are pathogens, being the cause of systemic mycoses of many organs, including the oral cavity [11]. Depending on the literature, the prevalence of fungal infections in healthy study volunteers ranges from 20% to 60%. In addition, it has been proven that fungi in the oral cavity in children and adolescents are detected twice as often as in healthy adults [12]. The most common oral yeast fungus is *Candida albicans*. It is also considered the main causative agent of oral candidiasis. The recently conducted systematic review showed *Candida* colonies increase, especially *C. albicans* species, during the first month of therapy with removable orthodontic appliances, followed by a decrease after a few months [13]. Less commonly described are *C. crusei*, *C. glabrata*, *C. tropicalis*, *C. parapsilosis*, *C. famata*, *C. pseudotropickalis*, and *C. dubliniensis*. These fungi can reside in the oral cavity for a long time without producing any disease symptoms. However, when the body’s homeostasis is disturbed and unfavorable conditions exist, they can cause risk to the health and sometimes even to the life of their host. Most of the described cases of oral candidiasis involve patients using prosthetic restorations and orthodontic appliances—mainly removable—as well as patients burdened with immune-compromised diseases, including AIDS or autoimmune diseases [14,15]. The pathogenic properties of *C. albicans* are manifested by its antigenic and morphological variability, ability to adhere to vascular endothelium, immunomodulatory activities, and production of lytic enzymes.

A healthy body benefits from a multitude of defense mechanisms that protect it from *Candida* expansion. These include a continuous process of exfoliation of epithelium, the flow of saliva and the organic and inorganic components it contains, the pH of the mucous membranes and skin, the coexistence of saprophytic bacteria competing for a place in the host cells or, finally, an efficient immune system. Superficial colonization of the oral cavity by the fungus is conditioned by its overcoming local environmental conditions, such as the salivary barrier or the continuity of the mucosa. Once the microorganisms penetrate inside the tissues, the colonization transforms into an infection. Local factors that increase the risk of oral candidiasis include changes in the composition of the microflora, a decrease in salivary flow, a diet rich in carbohydrates, and thinning and/or ulcerated epithelium resulting from the use of not-fitting dental restorations or orthodontic appliances. Moreover, regarding systemic factors, immunosuppression, prolonged antibiotics use, endocrine disruption, and folic acid and iron deficiencies should be mentioned [12,16]. 

In addition, the patient should be instructed on the necessity and importance of proper oral hygiene. Local irritants should be eliminated and properly selected vitamin supplementation should be implemented to correct deficiencies [17]. There are numerous publications in the literature on the effect of acrylic restorations on the adult population. Kozak et al. studied the effect of fixed orthodontic appliances on the distribution of dental biofilm [18]. However, few studies in this area concern removable orthodontic appliances used by the youngest patients [19,20,21]. 

Given the paucity of research on *Candida* species in children undergoing orthodontic treatment with removable appliances, the aim of this study was to evaluate the impact of orthodontic treatment with removable appliances on the growth of *Candida* spp. 

## 2. Materials and Methods

This study was conducted in accordance with the guidelines of the Helsinki Declaration and the protocol was approved by the Local Bioethics Committee of the Pomeranian Medical University in Szczecin, Poland (protocol number: KB-0012/55/17). Parents of participating patients gave written consent for their children to participate in the research project. At the same time, they were informed in detail about the type of research, the technique used to perform it, and the possibility of opting out of participation in the study at any stage.

### 2.1. Characteristics of the Study Group

The study included 60 patients (30 girls and 30 boys) of the Department of Orthodontics at the Pomeranian Medical University in Szczecin, Poland, aged 6 to 12 years, who were qualified for orthodontic treatment with removable appliances; Schwarz active plate (upper and/or lower). All the subjects had to meet all of the inclusion criteria, and individuals who did not meet all of the listed inclusion criteria were excluded from the study. The following inclusion criteria were applied: malocclusion, no syndrome diagnosis, female and male gender, no coexistence of systemic diseases, no drugs taken that could affect the results, also without taking antibiotics for 6 months before the start of the study, no history of trauma or surgical treatment in the orofacial region, no previous orthodontic treatment, and voluntary consent to participate in the research. Patients with good/optimal oral hygiene (<40%) as assessed by the approximal plaque index (API) were included in the study. Subjects with systemic diseases, taking medications that could affect the research outcomes, taking antibiotics 6 months or less before the start of the study or during the research, a history of trauma or surgery in the orofacial area, previous orthodontic treatment, as well as subjects with abnormal lip seal, mouth breathing, and/or individuals who were mentally unable to participate in the study procedures were excluded from the group. Decayed, missing, and filled teeth (DMFT) in permanent dentition and decayed, extraction-needed, and filled teeth (dmft) in the primary dentition index value > 6 and/or average/poor oral hygiene according to the API were criteria for excluding a patient from participating in the study. Both patients and their parents were instructed about the requirement to use the devices for 14–16 h a day, in order to achieve the best therapeutic results. Patients were advised to attend follow-up appointments every 4–5 weeks. 

The results of the subject and physical examination of the participating patients were recorded in the orthodontic examination card. In addition, due to the need to include additional data obtained for the purpose of this research project, a special patient examination card was created. The card recorded the results of microbiological cultures—the appearance of Petri dishes with Sabouraud’s medium after an appropriate incubation period and the results of fungal identification tests.

### 2.2. Collection of Samples and Preparation and Isolation of Candida colonies, Identification of Candida Species by VITEK^®^2 YST 

The following tests were performed on each patient on the day of handing out the devices (T1) and 6 months after the onset of treatment (T2):

1. Taking swabs from the oral cavity including the hard palate, buccal mucosa, and tongue using sterile swab sticks with a transport medium;
2. Performing cultures on Sabouraud’s medium;
3. Identification of fungal colonies was performed using the VITEK^®^2 YST automated card system (bioMérieux, Warszawa, Poland).

During the intraoral examination, a dental diagram was completed. The degree of loosening of deciduous teeth, the presence of carious cavities, and the degree of attrition of both deciduous teeth (physiological attrition) and permanent teeth (pathological attrition) were also noted. The dental status index for permanent teeth (DMFT) and deciduous teeth (dmft) was calculated. Oral hygiene was assessed during the examination using the API. The canine classes and first angle classes were initially assessed, as well as the amount of overbite and overjet. The final verification of the obtained results was carried out on the diagnostic cast of each patient. Each patient’s records consisted of an analysis of casts; a panoramic radiograph; and extraoral and intraoral photographs documenting the condition of the teeth, periodontium, and surrounding tissues, which also served as study material. When handing over the removable orthodontic appliance, detailed instructions on oral hygiene and appliance care were given. A brushing technique based on the principle of sweeping (roll) was recommended. All patients were advised to brush their teeth twice a day with fluoride toothpaste (1450 ppm) for at least 4 min. In younger children and those with less manual dexterity, it was recommended that an adult monitor the brushing process each time and repeat the brushing activity if necessary. Each child’s toothbrush was individually selected in terms of bristle hardness and length of the brush’s working part, taking into account, among other things, the child’s age, size of the mouth, and teeth. Flossing was suggested to ensure proper hygiene in the interdental spaces, especially in young patients with crowded teeth. In addition to verbal instruction, phantom models were used during the instruction to better illustrate a proper brushing and flossing technique.

Oral swabs were taken with a 15-cm-long stick tipped with a sterilized swab. Opening of the package containing the tube with the swab stick was performed directly at the patient. The swab was taken from the base of the tongue, buccal mucosa, and hard palate by gently rubbing the test site for 3 s. All swabs were taken early in the morning, after a 12 h fast. After collection, the swab stick was immediately placed in a tube of AMIES transport medium, signed with the code number assigned to each patient and placed in the transport refrigerator. Immediately after swab collection, the tube was transported to the Department of Microbiology at the Pomeranian Medical University in Szczecin, Poland. Sabouraud’s solid agar medium with gentamicin and chloramphenicol (bioMérieux, Warszawa, Poland) in the form of round amber-colored Petri dishes was used for the isolation and culture of fungi. Sabouraud’s medium was prepared in accordance with the recommendations for the composition of medium C in the European Pharmacopoeia. The main components included in the medium were peptones, glucose, and agar. The medium on the dishes was stored at a temperature of 2 to 8 degrees Celsius. Using a sterile inoculation loop, material was taken from the swab stick. Then, a small amount of the test material was spread with a zigzag over the entire surface of the agar medium located in the Petri dish and placed at 34 degrees Celsius. Incubation of the material lasted 48 h, and the culture was viewed daily. After incubation, further observation of microbial colony growth was conducted. In order to correctly identify yeast species, it was decided to use the VITEK^®^2 YST automated card system (bioMérieux, Warszawa, Poland). Each card contains 64 special wells and 47 fluorescent biochemical tests and is designed for single use. It detects more than 50 species of pathogenic yeast fungi. 

### 2.3. Statistical Analysis

The results of the study were statistically analyzed using STATISTICA 10 (StatSoft, Kraków, Poland) computer programs. The Shapiro–Wilk W test was used to verify that the results of the study conformed to a normal distribution. In order to verify the hypothesis regarding the existence and nonexistence of differences between the mean values for the independent variables, the Mann–Whitney U test and Student’s *t*-test of the independent variables were used. Spearman’s r-rank correlation coefficient and Pearson’s r-linear correlation coefficient were used to assess correlations between variables. To assess the relationship between qualitative or step variables, the chi^2^ test of independence with Yates’s correction or Fisher’s exact test was used. All results whose significance level *p* was less than 0.05 (*p* < 0.05) were considered statistically significant.

## 3. Results

The average follow-up time for treatment of children in the study group was 6.118 ± 0.212 months, with no statistical significance between girls and boys. The mean age of all patients participating in the study at the start was 9.02 ± 1.38 years. Given that the subsequent study was to be conducted approximately 6 months after the first study, the mean age of the subjects increased to 9.53 ± 1.39 years. The mean age of the girls at T1 was 8.83 ± 1.29 years, while that of the boys was 9.21 ± 1.47 years. The age of the patients at T2 changed—this was due to the requirements of the experiment being conducted. The mean age for girls was 9.34 ± 1.29 years, and for boys 9.72 ± 1.47 years. The difference in the mean age of the subjects at T1 and T2 between girls and boys was not statistically significant.

Swabs taken from the oral cavity allowed an overall assessment of the presence or absence of fungal colonization in the study group of patients. At the first examination (T1), 42 subjects, representing 70% of the study group, were free of *Candida*, while after 6 months of treatment, the number decreased to 25 (41.67%) (Table 1). In 17 subjects (28.33%), who initially showed no evidence of fungi in the mouth, the appearance of *Candida* strains was observed (Table 1).

Of the 18 subjects who were found to be colonized with fungi in their mouths during the T1 examination, the most commonly identified strain was *Candida albicans*—13 subjects (72.22% of all identified fungi). The study at T2 also showed that *C. albicans* was the fungus most frequently colonizing the oral cavity of 23 persons (38.33%). Three new strains *C. dubliniensis*, *C. kefyr*, and *C. krusei* were identified at T2 (Table 1).

Table 1 shows the percentage results of the occurrence of specific fungal species according to the time at which the study was conducted. Based on the analysis of the results obtained, there was a correlation between the microorganisms present in the second examination and orthodontic treatment with removable appliances (Rs = 0.29, *p* = 0.001). There were significant differences in the percentage distribution of specific fungal species depending on the time of examination. In addition, the prevalence of each type of *Candida* was analyzed. At T2, a statistically significantly less frequent negative culture result was obtained (*p* = 0.015), while *Candida albicans* (Rs = 0.18, *p* = 0.046) and *Candida dubliniensis* (Rs = 0.21, *p* = 0.022) were statistically significantly more frequently identified. 

In the first examination (T1), no patient showed more than 50 colonies in the preparation, 20% showed single colony growth, while 10% showed moderately abundant colony growth. The examination performed at T2 showed abundant fungal colony growth (>50 colonies in the culture) in 12 patients (20%) (Table 2). 

An analysis of the relationship between the number of fungal colonies grown in the preparation and the time at which the study was conducted showed a tendency for the number of fungal colonies to increase over time (Table 2). A statistically significant correlation was observed between fungal colony growth and orthodontic treatment with removable appliances. Analysis of the relationship between the number of isolated fungal colonies and the use of removable plates showed a statistically significant percentage increase in the number of these colonies at T2 (Rs = 0.37, *p* = 0.0003). 

### 3.1. Analysis of the Relationship between the Results of Fungal Colonies and Gender of Patients

Statistical analysis showed significant correlations between the number of isolated fungal colonies in both girls (Rs = 0.41, *p* = 0.001) and boys (Rs = 0.33, *p* = 0.0105) (Table 3). We also analyzed how specific categories of study variables differed from each other. 

The subgroups of girls and boys had fairly similar results for the type of fungi isolated. In the study at T1, 21 girls and 21 boys had negative culture results. In a follow-up study, conducted after six months, there were more positive results in both subgroups, with girls having slightly more positive results than boys. *Candida albicans* was the most common fungus isolated in both subgroups (Table 4).

### 3.2. Analysis of the Relationship between the Results of Fungal Cultures and the Age of the Patients

There was an overall increase in the number of *Candida* fungal colonies in both the younger and older patient groups. In the group of patients aged 9 years and older, there was a statistically significant increase in the number of fungal colonies with a *p* = 0.005 (Table 5).

Table 6 shows the results of the prevalence of specific fungal species according to the time at which the study was conducted in children in two age groups—the first for younger children and the second for older children. In both groups, a statistically significant relationship was observed between the detection of more fungal species and the use of removable appliances in the group of younger children (Rs = 0.28, *p* = 0.031) and in the group of older children (Rs = 0.31, *p* = 0.017).

In addition, individual types of *Candida* were analyzed quantitatively (Table 6). At T2, older children were statistically significantly less likely to have a negative culture result (Rs = −0.34, *p* = 0.009). Older patients had significantly more positive fungal cultures. Additionally, in the subgroup of children aged 9 years and older, statistically significantly more often *Candida albicans* fungi were isolated from collected samples (Rs = 0.27, *p* = 0.035).

## 4. Discussion

The oral cavity is a place where many types of microorganisms—from bacteria and viruses to fungi—coexist, as confirmed by the work of numerous researchers [22,23]. Some of these microorganisms are part of the normal oral microbiome and do not cause any negative effects in the host body. Among many researchers, there is no consensus on which group the fungi inhabiting the oral cavity should be classified in. Some scientists believe that they are part of the natural microbiome and do not have a destructive effect on the host body. From this point of view, the relationship between humans and fungus is typical commensalism, so the existence of the fungus in the oral cavity brings neither loss nor benefit to the host. On the other side of the scale are scientists for whom any number, even of single fungal colonies in the human body is a typical example of parasitism, i.e., an interaction on the basis of which the host suffers losses associated with the presence of a foreign genome [24]. Therefore, in the presented research, it was decided to check how often fungi colonize the oral cavity of children in whom there is no clinical manifestation of infection. According to our own research, the oral cavity of the majority of children presenting to the Orthodontics Clinic in Szczecin for orthodontic treatment was not colonized by any type of fungi. Of the 60 patients participating in the study, only 18 patients had positive cultures for fungi, accounting for 30% of all subjects. Among the positive results, more than 70% of the fungi were *Candida albicans*. In most of the children tested, the *Candida* spp. detected were single colonies of no more than nine colonies in the specimen. 

However, analyzing the results obtained six months after the beginning of treatment, there is a reversal trend of the predominance of negative results over positive ones. In our study, positive results were obtained in 58.33% of the participants, indicating the detection of fungal colonies in the analyzed material, which shows an almost twofold increase in positive results compared to the study before the start of treatment. The most frequently detected fungal species was *Candida albicans*. This was the result of almost 66% of positive cultures. According to our own observations, there was an increase in the number of colonies in the preparation after six months of treatment—most of the positive patients had a moderately abundant colony count, which meant the presence of 10 to 50 fungal colonies in the analyzed preparation. The first references in the scientific literature relating to changes in *Candida* count date back to 1979. Arendorf and Walker studied the relationships regarding the appearance of fungi in the oral cavity in patients using dental restorations and orthodontic appliances. The authors observed a significant increase in the number of fungi after six months of treatment with removable appliances, both on the surface of the teeth and on the oral mucosa [25]. In contrast, in 1982, a study by Addy et al. showed no statistically significant differences between groups of healthy adolescents without orthodontic treatment, with fixed orthodontic appliances, and treated with removable appliances. *Candida* carriage was demonstrated in 46–52% of subjects regardless of the group. However, the researchers noted a significant increase in fungal counts in those who had previously been diagnosed with *Candida* carriage and were under orthodontic treatment compared to untreated subjects. The authors of the study suggested that fixed and removable appliances may stimulate *Candida* proliferation in those who are *Candida* carriers; however, they did not determine whether fungal-free patients may develop an infection initiated by orthodontic treatment [20]. The first long-term study on the effects of removable appliances on fungal development was conducted in 1985 by Arendorf and Addy [21]. The authors observed 33 British youths between the ages of 8 and 17, varying in gender and age. They proved that before starting orthodontic treatment, 39% of the patients were *Candida* carriers, while after nine months, the number increased to 79%. Thirteen of the non-carrier patients showed the appearance of *Candida* during the course of treatment, of which only three patients still had fungal colonies in their mouths after orthodontic therapy. Two of the patients who were carriers of *C. albicans* before the start of treatment had negative culture results after the end of treatment, indicating that even single fungal colonies had not been isolated. Arendorf and Addy proved that there is a close dependence between acrylic removable appliances and the emergence of *Candida*. According to the authors, these appliances temporarily change the microbial status in the oral cavity [21]. The results of our study confirm the findings regarding the increase in the number of patients with oral fungal infection during orthodontic treatment with removable appliances. Moreover, similar results were obtained by Khanpayeh et al. [26]. In the studied group of 40 patients (21 female and 19 male) with an average age of 11.7 years and treated with removable appliances for about 9 months, 77.5% of positive cultures for *Candida* were obtained. In 9 patients, not even a single fungal colony was detected, which accounted for 22.5% of all the subjects. The authors published the results of microbiological cultures including the type of fungi isolated. The vast majority of the detected fungi were *Candida albicans* (62.5%). In addition, *C. tropicalis* (7.5%), *C. parapsilosis* (5%), and *C. krusei* (2.5%) were also isolated. Consistent with the results of our research, a similarly dominant group of fungi was *C. albicans*, which was detected in 38.33% of the subjects. The second most prevalent was *C. dublienensis,* isolated in 8.33% of individuals, followed by *C. parapsilosis* (6.67%), *C. kefyr* (3.33%), and *C. krusei* (1.67%). Comparing the results of Khanpayeh et al. and our study, we can risk the thesis that removable appliances predispose the patient to the development of *Candida*, with *C. albicans* being the most common species. Moreover, in a study conducted in Mexico, an increase in *Candida* spp. occurred after 4 weeks of treatment with removable appliances and was found in oral mucosa material in 30.9% of patients [19]. Additionally, in the study by Arika et al., both fixed and removable space maintainers caused an increase in the number of microorganisms in the oral cavity [27]. As confirmed by various analyses, the slight differences between studies regarding the prevalence of other types of fungi may be due to microbiological variability in the patient groups studied, which may be influenced by the latitude where the study was carried out [28,29,30].

In the conducted study, it was shown that positive *Candida* cultures before the start of treatment were at the same level regardless of gender. Six months after the start of treatment, there was a greater increase in positive *Candida* cultures in girls than in boys. When analyzing the culture results obtained in relation to the age of the patients, it can be seen that negative results predominated in children under nine years of age, both before and after the start of treatment. The general population trend in our study was maintained. Although the number of negative T2 tests decreased in this age group, it was still higher than the number of positive tests. In the group of older patients, 9 years and over, there were more negative results at T1, while there were more positive results at T2. From the review of the literature, it appears that there are no scientific reports that take into account differences between the age and gender of the subjects. It would be advisable to continue research in this area, because of the resulting possibility of learning more about groups at increased risk of contracting fungal infections during orthodontic therapy. In addition, our study showed that there were no statistically significant correlations between the type of fungi isolated and the gender and age of the patients studied. In our own material, the group of patients consisted of 30 female and 30 male patients. These relationships were quite similar with respect to the work of other researchers on orthodontic treatment in children, with girls more often being the predominant patient group [31,32,33]. In contrast, in analyses carried out in Brazil and Italy [34,35], both the groups of patients treated with fixed and removable appliances were predominantly boys. The significant predominance of female patients in most studies may be due to the fact that in girls, from an early age, more importance is placed on appearance, facial aesthetics, and smiling [35]. In order to allow multiple comparisons of the results obtained, depending on the gender and age differences of the study group, in this study it was decided to take both of these factors into account when analyzing the impact of removable appliances on the parameters studied. 

The results presented by other researchers largely coincide with those of our own study. Various authors have analyzed not only removable appliances as hypothesized to affect the oral microbiome, but also other removable prosthetic restorations [36,37]. The consideration of removable appliances and removable prosthetic restorations at the same time is justified by the fact that, in both cases, the acrylic plate can obstruct the flow of saliva between the plate and the oral mucosa in a significant way, which definitely impedes the self-cleaning process. In addition, an increase in temperature is observed beneath the mucosa-adherent surface of the denture plate or orthodontic appliance. This factor is considered to be conducive to the growth and proliferation of microorganisms, especially various types of fungi. Another issue often raised by researchers is the presence of micro-leaks and pores in the acrylic device, which combined with a warm and moist oral environment, may promote the growth of pathogens. In addition, the long hours of use of appliances and dentures promote micro-trauma to the oral mucosa, making it more vulnerable to fungal penetration and colonization of its deeper layers. The eradication of *Candida* spp. from the infected oral cavity is significantly hindered [37]. 

In our study, no clinical manifestations of fungal infections were observed. None of the patients developed full-blown oral candidiasis and did not show the typical symptoms that can accompany this disease, such as pain, itching, burning or differences in taste perception [38,39]. In spite of this, the results regarding candida colony number in the swabs taken show a significant increase over time. The increase in the number of oral fungal infections in the absence of clinical symptoms of candidiasis can be explained by the hypothesis of a high resistance of young, healthy organisms to the development of pathogenic microorganisms. The theory of commensal interdependence between fungus and human seems to be closest to the truth. As long as the host organism is characterized by intrinsic homeostasis and its well-being is not disturbed, the oral co-occurring pathogen brings neither loss nor benefit to the host [40,41,42,43]. Our results are consistent with a study by Hibino et al. where no healthy patient developed *Candida* infection as a result of orthodontic treatment. On the other hand, some non-*Candida* carriers appear to have converted to *Candida* carriers after the use of the appliance as a result of an unknown mechanism [15]. However, according to other researchers, when immunity declines and the organism’s internal balance is disturbed, commensalism can be replaced by parasitism. There is an intensive proliferation of microorganisms, manifested by the appearance of local clinical signs of infection. When the organism’s immunity further declines, the fungi can occupy further tissues and organs, resulting in the development of systemic mycosis [41,44,45].

In conclusion, the results of this study demonstrate that removable orthodontic appliances may adversely affect the oral cavity environment and disrupt its stable ecosystem. In many patients, this manifests itself as an increase in fungal colonies. The undesirable effects of removable appliances must therefore always be taken into account in the planning of orthodontic treatment and, as far as preventive measures are available, be efficiently counteracted. During orthodontic treatment, special attention should be paid to oral hygiene. It is important to keep in mind the limitations of the study, such as the small number of participants taking part in the study, and the limited age range of the participants while remembering that the problem has not been widely studied before. We are currently studying several of these topics. Further research is required on a larger group of subjects to confirm these study results.

## 5. Conclusions

1. Orthodontic treatment with removable appliances contributes to an increase in the colonization of the oral cavity by *Candida* spp. Fungi;
2. *Candida albicans* is the most commonly found in patients undergoing orthodontic treatment with removable appliances;
3. Patients in the older age group over 9 years old are more vulnerable to oral colonization by *Candida* spp. fungi in comparison to patients younger than 9 years old.

## Figures and Tables

**Table 1:** Relationship between the prevalence of each type of *Candida* to the time at which the study was conducted.

Type of *Candida*	T1		T2		Total No.	Chi^2^ *p*-Value	Fisher’s *p*-Value	R Rank	Spearman’s *ρ* *p*-Value
	No.	%	No.	%					
Absence	42	70.00	25	41.67	67	0.002	0.003	−0.29	0.002
*C. albicans*	13	21.67	23	38.33	36	0.046	0.072	0.18	0.046
*C. parapsilosis*	3	5.00	4	6.67	7	0.696	1.000	0.04	0.699
*C. tropicalis*	1	1.67	0	0	1	0.315	1.000	−0.09	0.319
*C. lusitaniae*	1	1.67	0	0	1	0.315	1.000	−0.09	0.319
*C. dubliniensis*	0	0	5	8.33	5	0.022	0.057	0.21	0.022
*C. kefyr*	0	0	2	3.33	2	0.153	0.495	0.13	0.156
*C. krusei*	0	0	1	1.67	1	0.315	1.000	0.09	0.319
Total	60	100.00	60	100.00	120				
Pearson’s chi^2^ test	17.23		df = 7		*p* = 0.015				
Spearman’s *ρ*	0.29		t = 3.344		*p* = 0.001				

**Table 2:** Analysis of the relationship between the number of isolated *Candida* colonies and the time at which the study was conducted.

No. of Candida	T1		T2		Total No.	Chi^2^ *p*-Value	Fisher’s *p*-Value	R Rank	Spearman’s *ρ* *p*-Value
	No.	%	No.	%					
Absence	42	70.00	25	41.67	67	0.001	0.003	−0.29	0.002
1–9 colonies	12	20.00	7	11.67	19	0.211	0.317	−0.11	0.214
10–50 colonies	6	10.00	16	26.67	22	0.018	0.032	0.22	0.018
>50 colonies	0	0	12	20.00	12	0.0002	0.0002	0.33	0.0002
Total	60	100.00	60	100.00	120				
Pearson’s chi^2^ test	22.17		df = 3		*p* = 0.0001				
Spearman’s *ρ*	0.37		t = 4.3177		*p* = 0.0001				

**Table 3:** Analysis of the relationship between fungal colony results and patient gender.

		No. of *Candida*	T1		T2		Total No.	Chi^2^ *p*-Value	Fisher’s *p*-Value	R Rank	Spearman’s *ρ* *p*-Value
			No.	%	No.	%					
		Absence	21	70.00	11	36.67	32	0.009	0.019	−0.33	0.009
Girls		1–9 colonies	6	20.00	5	16.67	11	0.738	1.000	−0.04	0.743
		10–50 colonies	3	10.00	7	23.33	10	0.165	0.298	0.18	0.171
		>50 colonies	0	0	7	23.33	7	0.004	0.010	0.36	0.004
		Total	30	100.00	30	100.00	60				
		Pearson’s chi^2^ test	11.82		df = 3		*p* = 0.008				
		Spearman’s *ρ*	0.41		t = 3.421		*p* = 0.001				
Boys		Absence	21	70.00	14	46.67	35	0.066	0.115	−0.24	0.068
		1–9 colonies	6	20.00	2	6.67	8	0.128	0.254	−0.20	0.133
		10–50 colonies	3	10.00	9	30.00	12	0.052	0.104	0.25	0.054
		>50 colonies	0	0	5	16.67	5	0.019	0.052	0.30	0.019
		Total	30	100.00	30	100.00	60				
		Pearson’s chi^2^ test	11.40		df = 3		*p* = 0.009				
		Spearman’s *ρ*	0.33		t = 2.643		*p* = 0.0105				

**Table 4:** The results of the prevalence of specific fungal species according to the time at which the study was conducted and the gender of the patients.

	Type of *Candida*	T1		T2		Total No.	Chi^2^ *p*-Value	Fisher’s *p*-Value	R Rank	Spearman’s *ρ* *p*-Value
		No.	%	No.	%					
Girls	Absence	21	70.00	11	36.67	32	0.009	0.019	−0.33	0.009
	*C. albicans*	6	20.00	11	36.67	17	0.152	0.252	0.18	0.157
	*C. parapsilosis*	2	6.67	2	6.67	4	1.000	1.000	0.00	1.000
	*C. tropicalis*	1	3.33	0	0	1	0.313	1.000	−0.13	0.321
	*C. lusitaniae*	0	0	0	0	0	1.000	1.000	-	-
	*C. dubliniensis*	0	0	3	10.00	3	0.075	0.237	0.23	0.077
	*C. kefyr*	0	0	2	6.67	2	0.150	0.491	0.19	0.155
	*C. krusei*	0	0	1	3.33	1	0.313	1.000	0.13	0.321
	Total	30	100.00	30	100.00	60				
	Pearson’s chi^2^ test	11.60		df = 7		*p* = 0.115				
	Spearman’s *ρ*	0.35		t = 2.852		*p* = 0.006				
Boys	Absence	21	70.00	14	46.67	35	0.067	0.115	−0.24	0.069
	*C. albicans*	7	23.33	12	40.00	19	0.165	0.266	0.18	0.171
	*C. parapsilosis*	1	3.33	2	6.67	3	0.553	1.000	0.08	0.561
	*C. tropicalis*	0	0	0	0	0	1.000	1.000	-	-
	*C. lusitaniae*	1	3.33	0	0	1	0.313	1.000	−0.13	0.321
	*C.dubliniensis*	0	0	2	6.67	2	0.150	0.491	0.19	0.155
	*C. kefyr*	0	0	0	0	0	1.000	1.000	-	-
	*C. krusei*	0	0	0	0	0	1.000	1.000	-	-
	Total	30	100.00	30	100.00	60				
	Pearson’s chi^2^ test	6.05		df = 7		*p* = 0.534				
	Spearman’s *ρ*	0.24		t = 1.857		*p* = 0.068				

**Table 5:** Analysis of the relationship between fungal colony results and patient age.

		No. of *Candida*	T1		T2		Total No.	Chi^2^ *p*-Value	Fisher’s *p*-Value	R Rank	Spearman’s *ρ* *p*-Value
			No.	%	No.	%					
		Absence	24	80.00	17	56.67	41	0.052	0.009	−0.25	0.053
<9 years old		1–9 colonies	5	16.67	2	6.67	7	0.227	0.423	−0.16	0.234
		10–50 colonies	1	3.33	5	20.00	7	0.044	0.103	0.26	0.045
		>50 colonies	0	0	5	16.67	5	0.019	0.05	0.30	0.019
		Total	30	100.00	30	100.00	60				
		Pearson’s chi^2^ test	11.05		df = 3		*p* = 0.011				
		Spearman’s *ρ*	0.31		t = 2.513		*p* = 0.014				
≥9 years old		Absence	18	60.00	8	26.67	26	0.009	0.018	−0.34	0.008
		1–9 colonies	7	23.33	5	16.67	12	0.517	0.748	−0.08	0.526
		10–50 colonies	5	16.67	10	33.33	15	0.136	0.233	0.19	0.141
		>50 colonies	0	0	7	23.33	7	0.005	0.011	0.36	0.005
		Total	30	100.00	30	100.00	60				
		Pearson’s chi^2^ test	12.85		df = 3		*p* = 0.005				
		Spearman’s *ρ*	0.44		t = 3.697		*p* = 0.0005				

**Table 6:** Results of the incidence of each fungal species according to the time at which the study was conducted in children in two age groups.

	Type of *Candida*	T1		T2		Total No	Chi^2^ *p*-Value	Fisher’s *p*-Value	R Rank	Spearman’s *ρ* *p*-Value
		No.	%	No.	%					
<9 years old	Absence	24	80.00	17	56.6	41	0.052	0.094	−0.25	0.053
	*C. albicans*	5	16.67	7	23.33	12	0.518	0.748	0.08	0.526
	*C. parapsilosis*	0	0	1	3.33	1	0.312	1.000	0.13	0.321
	*C. tropicalis*	1	3.33	0	0	1	0.313	1.000	−0.13	0.321
	*C. lusitaniae*	0	0	0	0	0	1.000	1.000	-	-
	*C. dubliniensis*	0	0	3	10.00	3	0.075	0.237	0.23	0.077
	*C. kefyr*	0	0	2	6.67	2	0.150	0.491	0.19	0.155
	*C. krusei*	0	0	0	0	0	1.000	1.000	-	-
	Total	30	100.00	30	100.00	60				
	Pearson’s chi^2^ test	8.53		df = 7		*p* = 0.288				
	Spearman’s *ρ*	0.28		t = 2.204		*p* = 0.031				
≥9 years old	Absence	18	60.00	8	26.67	26	0.009	0.018	−0.34	0.009
	*C. albicans*	8	26.67	16	53.33	24	0.035	0.064	0.27	0.035
	*C. parapsilosis*	3	10.00	3	10.00	6	1.000	1.000	0.00	1.000
	*C. tropicalis*	0	0	0	0	0	1.000	1.000	-	-
	*C. lusitaniae*	1	3.33	0	0	1	0.313	1.000	−0.13	0.321
	*C. dubliniensis*	0	0	2	6.67	2	0.150	0.491	0.19	0.155
	*C. kefyr*	0	0	0	0	0	1.000	1.000	-	-
	*C. krusei*	0	0	1	3.33	1	0.313	1.000	0.13	0.321
	Total	30	100.00	30	100.00	60				
	Pearson’s chi^2^ test	10.51		df = 7		*p* = 0.161				
	Spearman’s *ρ*	0.31		t = 2.441		*p* = 0.017				

## Data Availability

The datasets used to support the conclusions of this article are included within the article. Access to other data will be considered by the corresponding author upon request.
